# Testing of a Model with Latino Patients That Explains the Links Among Patient-Perceived Provider Cultural Sensitivity, Language Preference, and Patient Treatment Adherence

**DOI:** 10.1007/s40615-015-0114-y

**Published:** 2015-06-20

**Authors:** Jessica D. Jones Nielsen, Whitney Wall, Carolyn M. Tucker

**Affiliations:** Department of Psychology, City University London, South Hampton Square, London, EC1V 0HB UK; Department of Psychology, Fayetteville State University, Fayetteville, NC USA; Department of Psychology, University of Florida, Gainesville, FL USA

**Keywords:** Treatment adherence, Latinos, Provider cultural sensitivity, Patient-provider interactions

## Abstract

**Introduction:**

Disparities in treatment adherence based on race and ethnicity are well documented but poorly understood. Specifically, the causes of treatment nonadherence among Latino patients living in the USA are complex and include cultural and language barriers.

**Purpose:**

The purpose of this study was to examine whether patients’ perceptions in patient-provider interactions (i.e., trust in provider, patient satisfaction, and patient sense of interpersonal control in patient-provider interactions) mediate any found association between patient-perceived provider cultural sensitivity (PCS) and treatment adherence among English-preferred Latino (EPL) and Spanish-preferred Latino (SPL) patients.

**Methods:**

Data from 194 EPL patients and 361 SPL patients were obtained using questionnaires. A series of language-specific structural equation models were conducted to test the relationship between patient-perceived PCS and patient treatment adherence and the examined mediators of this relationship among the Latino patients.

**Results:**

No significant direct effects of patient-perceived PCS on general treatment adherence were found. However, as hypothesized, several significant indirect effects emerged. Preferred language appeared to have moderating effects on the relationships between patient-perceived PCS and general treatment adherence.

**Conclusion:**

These results suggest that interventions to promote treatment adherence among Latino patients should likely include provider training to foster patient-defined PCS, trust in provider, and patient satisfaction with care. Furthermore, this training needs to be customized to be suitable for providing care to Latino patients who prefer speaking Spanish and Latino patients who prefer speaking English.

## Introduction

The causes of health disparities in treatment adherence are complex and often involve differential access to health care and language barriers among racial and ethnic groups living in the USA. Language, in particular for Latinos, in addition to lived experiences, beliefs, and values continues to be the biggest barrier to patient treatment adherence and have been associated with poor health outcomes in this group. To date, only a few studies have examined the role that language and patients’ perceptions in patient-provider interactions (i.e., trust in provider, patient satisfaction, and patient sense of interpersonal control in patient-provider interactions) play in low adherence rates among this population. One study identified an association between patients’ perceived lack of control during patient-provider interactions and patients’ reduced treatment adherence behaviors among a sample of Hispanic/Latina women [1]. Another study identified significant linkages between patients’ trust in their physician and their adherence to their physicians’ treatment recommendations [2]. There are also few studies that have considered the influence of language barriers on treatment adherence of Spanish-preferred Latino patients living in the USA. While some studies suggest that language barriers do not affect patients’ appointment follow-through or dropout rates [3, 4], other studies have found that patients who did not speak the same language as their health care providers were less likely to adhere to their medication regimen [5]. Additionally, it has been found that language barriers have a significant impact on the treatment adherence practices among Spanish-preferred Latinos [6].

It is noteworthy that it has been found that cultural factors such as perceptions of the cultural sensitivity of their health care providers’ behaviors and attitudes can impact the treatment adherence of these patients [7, 8]. Given that the majority of health care providers who serve the Latino patient population identify as non-Latino, Latino patients will often receive health care services from providers who do not speak Spanish and are not aware of or do not understand the social norms and cultural beliefs such as the value of *respeto* and *paternalismo* of any of the various Latino cultural groups. It has been asserted that patient-provider linguistic and cultural differences may lead to poor adherence to treatment regimens among Latino patients [9–11]. Given that multiple cultural and linguistic factors may be associated with treatment adherence among Latinos, more research is needed that examines these associations rather than research that examines the association of only one cultural or linguistic variable in association with treatment adherence that has often been done. Research that examines the associations of multiple cultural and linguistic factors with treatment adherence among Latinos has possibly been impeded by the lack of treatment adherence models for such research [12].

Tucker’s Patient-Centered Culturally Sensitive Health Care Model (PC-CSHC) model [13] has been recently been set forth as a culturally sensitive model for explaining treatment adherence and health outcomes among culturally diverse patients. According to the PC-CSHC model, patient-centered culturally sensitive health care promotes patent-perceived provider cultural sensitivity (PCS), which in turn promotes trust in and comfort with the provider, both of which positively influence patient satisfaction with health care received and patient-perceived interpersonal control in patient-provider interactions and reduce patient-reported physical stress. Additionally, according to the PC-CSHC model, patient satisfaction with care and patient-perceived interpersonal control in patient-provider interactions positively impact patients’ health behaviors, whereas patient-reported physical stress negatively impacts patients’ health behaviors.

**Fig. 1:**
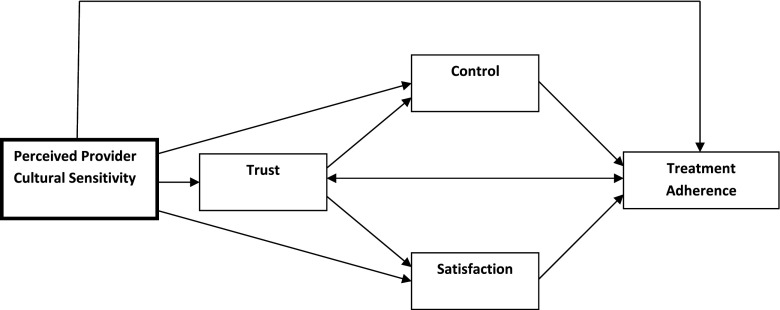
Modified patient-centered culturally sensitive health care (PC-CSHC) model

The aim of the present study is to provide an empirical test of a slightly modified version of the PC-CSHC model (Fig. 1) using multigroup structural equation modeling. It was hypothesized that (a) the most proximal and direct impact of patient-perceived PCS will be on patients’ perceived trust in their provider, satisfaction with their provider, and their perceived interpersonal control in patient-provider interactions; (b) patient-perceived PCS will also have indirect effects (through trust in their provider, satisfaction with their provider, and patient-perceived interpersonal control in interactions with their provider) on general treatment adherence; and (c) trust in provider, satisfaction with provider care, and patient-perceived control in patient-provider interactions will have direct effects on general treatment adherence. It was also hypothesized that the preferred language of the participating Latino patients would moderate these relationships. To test this second hypothesis, it was determined whether the model resulting from the structural equation modeling analysis fit equally well for English-preferred Latino (EPL) patients and Spanish-preferred Latino (SPL) patients.

## Methods

### Participants and Procedures

The present study was part of a larger national study on the characteristics of patient-centered, culturally sensitive health care. The procedures for this larger study are described in detail elsewhere [14]. Approval for the larger study was received from the Institutional Review Board (IRB) at the University of Florida.

In brief, the larger study participants were 1716 culturally diverse patients recruited from among patients who utilize any of 67 health care sites located in the Northeast (5 %), Midwest (10 %), South (30 %), and West (50 %) of the USA, in particular regions that were mostly populated by racial/ethnic minorities and low-income individuals. A small percentage (5 %) of sites did not report their geographic location. Overall, efforts were made to identify and recruit sites that were located in/around culturally diverse communities with disproportionately large percentages of African Americans/Black and Latinos—two groups that are more typically culturally different from their health care providers than Whites. Of the 67 health care sites, 71 % were community health care centers, 13 % were private practices, 7 % were health departments, 5 % were hospitals, and 4 % were other types of health care sites (e.g., halfway houses for rehabilitation). These patients were asked to anonymously complete an assessment battery that included measures of the variables in the slightly modified PC-CSHC model tested in the present study. Among the larger study sample were 588 (34 %) self-identified Latino patients—the patients that were used as the sample for the present study.

The aforementioned 588 Latino patients constituting the sample for the present study utilized health care services from among 48 of the 67 health care sites from which the patients in the larger study were recruited. Sixty percent of these 588 Latino participants indicated that they were born outside of the USA. Most of the participants were from Mexico (55 %), with 12 % from the Caribbean (Cuban, Dominican Republic, and Puerto Rico), 6 % from Central America (El Salvador, Guatemala, Honduras, Nicaragua, and Panama), and 6 % from South America (Brazil, Chile, Colombia, Ecuador, and Peru). The remaining 21 % of Latino sample did not specify their country of origin. Furthermore, 25 % were between the ages of 25 and 34, 65 % were female, and 57 % had equal to or less than a high school education. Latino patients were further classified in the present study into two language preference subgroups based on the language they preferred to complete their questionnaires: English-preferred Latino (EPL, 35 %) patients and Spanish-preferred Latino (SPL, 65 %) patients.

### Measures

In the larger study, patient participants were asked to anonymously complete an assessment battery (AB) packet consisting of 12 brief study questionnaires. Only six of these 12 questionnaires were specifically used to test the patient-provider interaction variables of the modified version of the PC-CSHC model. All patient questionnaires were translated into Spanish, independently back-translated by experienced translators, and then verified by certified translators to confirm translation reliability. Additionally, the reading levels of some of the items and some of the directions within the patient measures were slightly altered to make the inventories more easily understood by individuals with limited educational backgrounds and all questionnaires were reviewed for cultural appropriateness and validity.

The Patient Demographic and Health Data Questionnaire was constructed by the PI and her research team and was used to obtain general demographic and health information about each patient participant, such as age, race/ethnicity, gender, educational background, and immigration status. The questionnaire consists of 24 items. Sample items are “What is your age?” and “What is your gender?” “In general, how would you describe your health?” and “How many times each year do you see the health care provider that you see most often?”

The 129-item Tucker-Culturally Sensitive Health Care Inventory – Patient Form (T-CSHCI-PF) measures the level of self-reported patient-perceived cultural sensitivity in health care experienced, including perceived cultural sensitivity of providers’ and staff members’ behaviors and attitudes, and of the health care site environment and policies [14]. Recent use of the T-CSHCI-PF revealed it to have excellent internal consistency and test-retest stability, with a Cronbach’s alpha that exceeded 0.90 among an ethnically diverse sample that included English-speaking and Spanish-speaking Latinos [15]. With respect to T-CSHCI-PF items in the present study, only the scores for the Provider Behaviors and Attitudes subscale were used to measure level of patient-perceived provider cultural sensitivity. Cronbach’s alphas were 0.97 for the total Latino patient sample, 0.97 for the EPL patients, and 0.97 for the SPL patients. Tucker et al. [15] reported means and standard deviations for the T-CSHCI-PF that ranged from 3.11 (SD = 0.52) to 3.26 (SD = 0.54) for a community sample of African American patients, and means and standard deviations ranging from 2.94 (SD = 0.37) to 3.39 (SD = 0.50) for a community sample of non-Hispanic white American patients. All items on the T-CSHCI-PF are rated on a four-point Likert scale where 4 = “strongly agree” to 1 = “strongly disagree.” Scores for this questionnaire are averaged to yield a mean score for each subscale. Higher scores indicate greater self-reported levels of patient-perceived cultural sensitivity, whereas lower scores indicate lower self-reported levels of patient-perceived cultural sensitivity. Sample items from each T-CSHCI-PF subscale are “The health care provider I see most often when I visit my health care center or office understands my culture;” “The front office staff members at my health care center or office do not view patients of my race/ethnicity as uneducated and unable to read;” and “My health care clinic has official interpreters for patients who do not speak English.”

The Health Care Justice Inventory (HCJI) [16] is a ten-item scale that measures procedural and distributive justice in the health care context. Specifically, the HCJI consists of two subscales (Trust and Impartiality); however, for the purposes of this study, only the Trust subscale, which consists of five items, was used to measure the overall trust that the patient respondent has in his/her health care provider. Overall, the Trust subscale has high internal consistency (Cronbach’s alpha = 0.93) [16]. With respect to the present study, Cronbach’s alphas for the Trust subscale were 0.91for the total Latino patient sample, 0.89 for the EPL patients, and 0.92 for the SPL patients. No normative data for the HCJI could be found. All items on the scale are rated on a four-point Likert scale where 0 = “strongly disagree” to 3 = “strongly agree.” Subscale scores are obtained by summing up the item scores within each subscale. For the Trust subscale, higher scores indicate more trust perceived in their health care provider by the patient participant. Sample items from the HCJI Trust subscale are “You accept your health care provider’s decisions;” and “Your health care provider was honest with you.”

The Patient Satisfaction Questionnaire Short Form (PSQ-18) [17, 18] is an 18-item short-form version of the 50-item Patient Satisfaction Questionnaire III. The PSQ-18 is designed to measure patients’ attitudes toward characteristics of doctor and medical care services, and general satisfaction with health care received. The PSQ-18 has been reported to have a high internal consistency that exceeded 0.90 among population samples with various ethnic and racial groups which included Latino in the sample [15]. Cronbach’s alphas were 0.74 for the total Latino patient sample, 0.84 for the EPL patients and 0.62 for the SPL patients. It is important to note that the satisfaction in physician care measure (i.e., the PSQ-18) has a normative score based on a diverse group of study participants, including non-Hispanic white, African American, Hispanic/Latino/a, and Asian/Pacific Islander individuals; however, normative data is not available for each racial and ethnic group [19]. All items on the scale are rated on a five-point Likert scale where 1 = “strongly agree” to 5 = “strongly disagree.” Scores are obtained by averaging the items within each subscale. Higher scores indicate greater patient satisfaction within each health care dimension. Sample items from the PSQ-18 are “I am dissatisfied with some things about the medical care I receive;” and “My doctors treat me in a very friendly and courteous manner.”

The 18-item Patient-Practitioner Orientation Scale (PPOS) [20] was used to measure patients’ orientations and beliefs toward control in patient-provider interactions. Orientations, in this context, are relatively stable sets of personal beliefs and preferences about patient-provider interactions. Previous research has shown that the PPOS has satisfactory reliability (Cronbach’s coefficient alpha = 0.75 to 0.88) among a culturally diverse sample of patients which included Latino subgroups [16] and has demonstrated adequate validity [21, 22]. In the present study, Cronbach’s alphas for the PPOS were 0.79 for the total Latino patient sample, 0.76 for the EPL patients, and 0.76 for the SPL patients. No normative data for the PPOS could be found. The PPOS consists of two subscales (Sharing and Caring) with nine items each. All items on the scale are rated on a six-point Likert scale where 1 = “strongly disagree” to 6 = “strongly agree.” The Sharing subscale measures the degree to which the respondent believes that patients should take an active and participatory role in the health care decision-making process. The Caring subscale measures the degree to which the respondent sees the patient’s expectations, feelings, and life circumstances as crucial elements in the treatment process. Higher total scores and scores on the Sharing and Caring subscales reflect more patient-centered beliefs (sharing control, focus on the whole person), and lower scores reflect more physician-centered beliefs (high doctor control, focus on biomedical issues). Sample items from the PPOS Sharing and Caring subscales, respectively, are “It is often best for patients if they do not have a full explanation of their medical condition;” and “The patient must always be aware that the doctor is in charge.”

The five-item General Adherence Measure (GAM) is a self-report measure of treatment adherence and was constructed in the Medical Outcomes Study to summarize information about a patient’s general or typical tendency to adhere to medical recommendations, regardless of the type of treatment recommended [23, 24]. The internal consistency reliability of the scale was found to be acceptable (Cronbach’s coefficient alpha = 0.81), while the 2 years stability was *r* = 0.41 (DiMatteo et al. 1992). In the present study, Cronbach’s alphas for the GAM were 0.60 for the total Latino patient sample, 0.69 for the EPL patients, and 0.53 for the SPL patients. No normative data for the measures of general treatment adherence measure could be found. Total scores for the GAM are calculated by taking the average of responses to the five items and transforming the result linearly into a 0–100 distribution. Higher scores indicate more treatment adherence from the respondent. Sample items from the GAM are “I had a hard time doing what my provider suggested I do;” and “I found it easy to do the things my provider suggested I do.”

### Overview of Statistical Analyses

To test the modified PC-CSHC model, multigroup structural equation modeling (SEM) analyses were conducted by employing AMOS 17.0 program (SmallWaters Corp., Chicago, IL). This method simultaneously tests the effects of independent variables on various dependent variables in one model, as well as direct and indirect (mediating) effects. Furthermore, the fit of different models to the data can be compared; consequently, SEM is a particularly appropriate analytic method for testing the hypotheses set forth in the current study. Each model fit analysis was evaluated using multiple indicators of fit: the chi-squared (*χ*) index, the comparative fit index (CFI), the root mean square error of approximation (RMSEA), the normed-fit index (NFI), and the Tucker Lewis Index (TLI) [25, 26].

To determine if the tested modified PC-CSHC model was comparable for the language groups, the model was tested with simultaneous multigroup path analyses [27]. These types of analyses provide more powerful tools for testing the impact of language group differences by imposing factor invariance across the two language groups simultaneously [28]. The invariance of the measurement models across language groups was tested using full-information maximum likelihood (FIML) estimation under the assumption that data will be missing at random [29]. FIML has been selected in previous studies as an optimal method for handling missing data [30, 31].

**Fig. 2:**
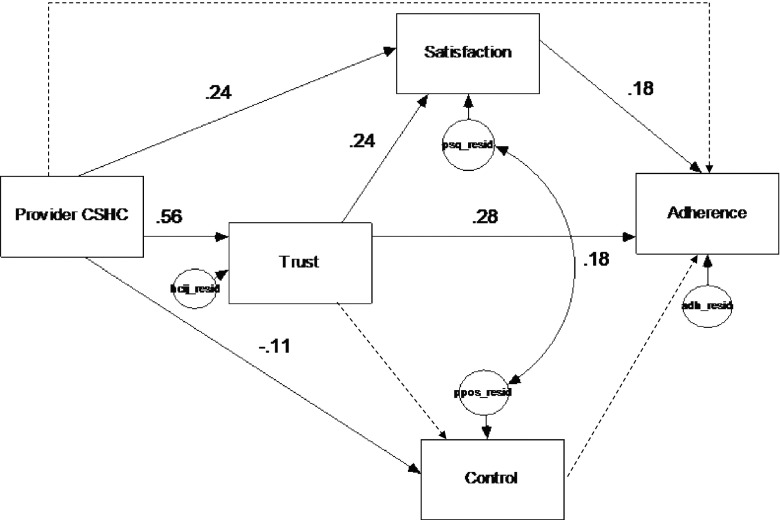
Standardized parameter estimates for full sample data (*n* = 555, all parameters had critical ratios >1.96)

A fully recursive model across the two language groups of patients was estimated using the proposed model (Fig. 2) by constraining all path coefficients (parameters) to be equal across both groups. Secondly, the path coefficients were freely estimated across groups. If the *χ*^2^ of the constrained model was significantly larger than the *χ*^2^ of the unconstrained model, the assumption of invariance would not be tenable. Specifically, chi-squared difference tests were used to compare these two models and to evaluate if, in general, the paths predicted in the theoretical model would differ across the language groups. The chi-squared index provides a test of the null hypothesis, which assumes that the reproduced covariance matrix has the specified model structure (i.e., that the model “fits the data”).

If the null hypothesis is “correct,” then the obtained chi-squared value should be small, and the *p* value associated with the chi-squared value should be relatively large (*p* < 0.05). If the omnibus chi-squared is not statistically significant, then it can be concluded that the same model can be applied to both groups. To detect which paths were different for the two language groups, each group’s path coefficients (parameters) were compared and assessed for statistical significance at the a priori α of 0.05. Once path coefficients that reached statistical significance were identified, the nonsignificant paths were eliminated by setting the parameters equal to zero to test whether a more parsimonious model would fit the data equally well. Again, the chi-squared difference test was used to evaluate the relative improvement or deterioration of the new model, and the two models using EPL and SPL data were compared. Lastly, a new and parsimonious model will be tested for each language group separately.

## Results

### Preliminary Analyses and Descriptive Statistics

Prior to the present study’s main analyses, exploratory data analyses were conducted to inspect the data for univariate normality, multivariate normality, outliers, multicollinearity, relative variances, and missing data. The results of this exploratory data analysis were used to determine whether the data met the criteria necessary for the planned statistical procedures [19]. Violations to the assumption of multivariate normality and relative variances were identified prior to conducting the planned study analyses. Nonparametric tests were conducted, and cases were deleted appropriately if data were nonnormal. Only two cases were identified through Mahalanobis distance as multivariate outliers with *p* < 0.001. Thirty-one cases were identified as univariate outliers with a z-score standard deviation greater than 2.95. With all 33 outliers deleted, 194 cases remained in the EPL group and 361 remained in the SPL group. Thus, data from 555 Latino patient participants were utilized in the analyses for the current study.

Table 1 presents the correlations, means, and standard deviations of the study variables separated by language preference groups. Pearson correlations were conducted to examine the associations among the major variables of interest in this study. When separated by language groups, general treatment adherence is correlated with all major variables of interest with the exception of the patient control of the treatment decision-making process variable (EPL *r* = 0.085 and SPL *r* = 0.094). Internal consistency for each major variable under investigation in this study is presented by language group in Table 1. Inter-item reliabilities were generally acceptable for research purposes (Cronbach’s alphas > 0.7). In the present study, the EPL patient participants had lower mean ratings on patient-perceived provider cultural sensitivity (*M* = 3.27, SD = 0.49) than did the SPL patient participants (*M* = 3. 32, SD = 0.46). Additionally, the EPL patient participants had slightly higher mean ratings of treatment adherence (*M* = 3.16, SD = 0.59) than did the SPL patient participants (*M* = 3.09, SD = 0.57).

**Table 1:** Correlations, means, and standard deviations of variables by language preference

		1	2	3	4	5	Mean	SD	α
English	1. Adherence	1.00	0.30**	0.085	0.23**	0.16*	3.16	0.59	0.69
	2. Trust		1.00	0.043	0.53**	0.55**	12.00	2.66	0.89
	3. Control			1.00	0.07	−0.02	4.15	1.02	0.76
	4. Satisfaction				1.00	0.54**	3.59	0.74	0.84
	5. PCS					1.00	3.27	0.49	0.97
Spanish	1. Adherence	1.00	0.327**	0.09	0.32**	0.18**	3.09	0.57	0.53
	2. Trust		1.00	−0.08	0.35**	0.56**	11.59	2.59	0.92
	3. Control			1.00	0.20**	−0.15**	3.77	0.99	0.76
	4. Satisfaction				1.00	0.34**	3.57	0.54	0.62
	5. PCS					1.00	3.32	0.46	0.97

Correlation coefficients with an * and ** are significant at the 0.05 and 0.01 level, respectively, according to a one-tailed test

*PCS* provider cultural sensitivity

### Model Fit

First, using the modified version of PC-CSHC model tested in this study, a fully recursive model was estimated across the full sample of Latino patients by constraining all path coefficients (parameters) to be equal across the two language groups. The constrained model yielded an acceptable level of fit for the two groups, *χ*^2^ (10, *N* = 194) = 25.978, CFI = 0.96, RMSEA = 0.05, NFI = 0.94, and TLI = 0.89, suggesting that patient-perceived PCS and patient-perceived patient-provider interaction factors are linked to general treatment adherence among the Latino patients. Figure 2 depicts the model tested in the full sample. The *R*^2^ values summarize the variation explained, and this variance was higher for trust in provider (30 %) and satisfaction with provider care (18 %) than for patient interpersonal control in the patient-provider interaction (1 %). The overall model explained 14 % of the variance in general treatment adherence using the full sample data. The standardized path coefficients (β) or parameters for the constrained model were significant (CR > 1.96), and show the magnitudes of the relationship between the different constructs. Figure 2 also shows that the model only included indirect effects on general treatment adherence.

**Fig. 3:**
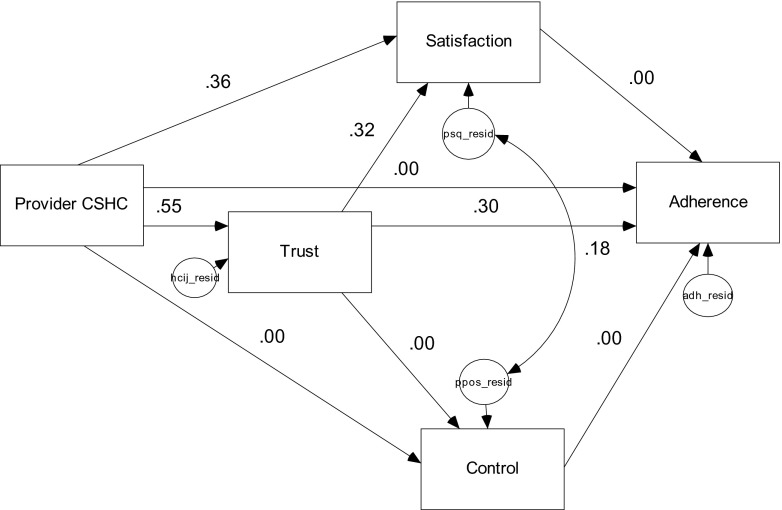
Standardized parameter estimates for English-preferred Latinos reduced model (*n* = 196, all parameters had critical ratios >1.96)

**Fig. 4:**
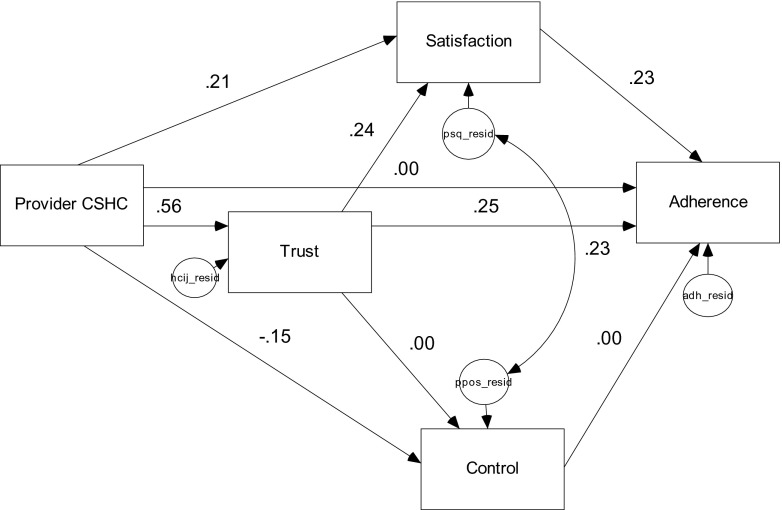
Standardized parameter estimates for Spanish-preferred Latinos reduced model (*n* = 361, all parameters had critical ratios >1.96)

To test if there were language group differences in the linkages between patient-perceived PCS and general treatment adherence—the parameters were freely estimated across language preference groups. The chi-squared difference test of differences between the two models supported the second hypothesis in that the parameters of the two language groups were significantly different from each other. Based on the fit indices, the freely estimated model provided a better fit of the data than the constrained model (see Table 2). To detect which paths were different for the two language groups, each group’s parameters were compared and assessed for statistical significance at the a priori alpha level of 0.05. The standardized parameters for the reduced model involving EPLs and the reduced model involving the SPLs are presented in Figs. 3 and 4, respectively.

**Table 2:** Goodness of fit indices for the model comparisons

Model	*df*	*χ* ^2^	RMSEA (90 % CI)	CFI	NFI	TLI
Model 1 (equal)	10	25.98	0.054 (0.029–0.080)	0.962	0.942	0.885
Model 2 (free)	1	2.61	0.054 (0.000–0.139)	0.996	0.994	0.885
Model 3 (reduced)	9	8.58	0.000 (0.000–0.046)	1.000	0.981	1.003
Model comparisons				*df*	*χ* ^2^ diff	*p* value
Models 1 and 2				9	23.37	*p* < 0.005
Models 2 and 3				8	5.97	0.650

## Discussion

As the population of Latinos in the USA continues to increase in the coming years, culturally and linguistically sensitive health care practices with this group will become more important to health care organizations, health professionals, and health policy makers. Current models of treatment adherence do not adequately address the unique needs of Latino patients living in the USA. The purpose of the current study was to test a modified version of Tucker’s PC-CSHC model among a sample of Latino patients. This study is the first study that has examined multiple cultural and linguistic predictors of general treatment adherence in a sample of research-identified Spanish-preferred Latinos and English-preferred Latinos living in the USA. Importantly, the sample included enough Spanish-speaking Latinos to enable meaningful analyses of the impact of language on the examined relationships, and it is anchored in a conceptual model (i.e., the PC-CSHC model) that included Latino patients in its development. The connection between patient-perceived provider cultural sensitivity and low treatment adherence among Latinos is complex and does not simply represent a language barrier. Therefore, understanding the cultural context in which diverse Latinos experience healthcare is critical to addressing low treatment adherence among this group.

As hypothesized, the path model analyses revealed significant links between patient-perceived PCS and general treatment adherence to the provider recommended treatment regimen, with some differences in association with language preference (English or Spanish). Overall, the findings provide support for the provision of patient-centered culturally sensitive health care and provide empirical support for use of the modified PC-CSHC model with Latino patients. Prior to this study, there was little empirical evidence of direct links between PCS and treatment adherence among Latino patients [32, 33].

Although the general tenets of the modified PC-CSHC model fit for both the English-preferred and Spanish-preferred patients in the present study, there were some notable model differences. Among both language preference groups, patient-perceived PCS had direct effects on important indicators of confidence and comfort with provider (i.e., trust and satisfaction with provider care), though the effect on satisfaction with provider care was stronger for the English-preferred patients. For the Spanish-preferred patients in this study but not English-preferred patients, patient-perceived PCS also had a direct negative effect on patient control in the treatment decision-making process. This may be because increasing PCS may represent the awareness of existing cultural barriers to health care and consequently patient-perceived lack of control over the patient-provider relationship. Trust in provider and patient-perceived interpersonal control in patient-provider interactions were also linked to satisfaction with provider care for both language groups. The size of the association between trust in provider and satisfaction with provider care for English-preferred patients was significantly larger than observed with the Spanish-preferred patients.

Tests of indirect effects revealed that, not surprisingly, for both language groups, satisfaction with provider care was likely an indirect function of the effect that patient-perceived PCS had on trust in provider. In essence, both groups were likely to have greater trust in providers if they deemed them to be culturally sensitive. In turn, that trust translated into greater likelihood of being satisfied with the care received. The indirect effects of patient-perceived cultural sensitivity were mediated only through trust in provider and satisfaction with provider care, and not through patient-perceived interpersonal control in patient-provider interactions. Additionally, no significant direct path between patient-perceived PCS and general treatment adherence was observed in the model. These findings suggest that Latino patients with higher levels of patient-perceived PCS tend to report higher levels of trust in provider and satisfaction with provider care, which in turn contribute to higher general adherence to provider recommended treatment regimen. Contrary to the stated hypothesis, no significant correlation was found between patient-perceived control in patient-provider interactions and general treatment adherence. Overall, these current results suggest that language differences and most patient-provider interaction variables may influence the treatment adherence among Latino patients. Language preference differences between Latinos are important, as well as patient-perceived cultural sensitivity, trust, satisfaction. However, control did not have a strong relationship with treatment adherence, and may demonstrate the problem of examining the direct relationship between these variables without considering provider variables or confounding factors.

The current study has important implication for improving treatment adherence among Latino patients. The preliminary evidence in this study that language moderates the relationship between patient-perceived cultural sensitivity in health care experienced and treatment adherence for both language groups, and the evidence that patient satisfaction and patient trust mediate the relationship between patient-perceived provider cultural sensitivity and treatment adherence highlight the need for interventions aimed at improving communication between health care providers and patients and provide support for interventions aimed at improving interpersonal processes of care between health care providers and Latino patients.

Understanding the predictors of general treatment adherence among various subgroups of Latino patients, such as the different language groups in the present study, is particularly important given the findings in the present study that the predictors of general adherence differ between patients who prefer to speak Spanish and patients who prefer to speak English. The above-identified linguistic differences in the model fit provide support for testing and evaluating culturally sensitive health care models, such as the PC-CSHC model, separately for patients who differ with regard to major demographic variables (e.g., language differences, country of origin). This approach is consistent with the difference model research approach that asserts that it is important to separately study groups that differ on major demographic variables, such as language differences, as there is no adequate means to statically control for these cultural differences [13].

### Limitations and Future Research

Despite the strengths of the present study, there are notable limitations that should be considered when interpreting the study findings. The first limitation is the generalizability of the study’s findings. Experiences of Latino patients living in the USA who access the health care system cannot be generalized from the findings of this study due to the fact that the language groups were limited to a modest sample size. Additionally, patients were not randomly selected to participate in the present study. As such, the present study should be replicated with a larger and randomly selected sample with a greater representation of Latino patients who are utilizing health care services.

Another limitation of the present study is the use of self-report measures, which raises questions regarding the reliability of the obtained data. Self-report measures of treatment adherence may encourage socially desirable responses rather than accurate responses [34]. Future studies similar to the present study should include a social desirability instrument, as data from it would enable controlling for social desirability. It is important to note that previous studies have concluded that self-reports of adherence behaviors are highly consistent with actual adherence as recorded in medical charts or insurance claims, even in low-income groups [35]. Future studies, however, may benefit from gathering data on patient treatment adherence from multiple sources, such as providers and family members.

Additionally, the cross-sectional design of the present study does not allow for observation of the variables of interest over time and does not allow determination of causal relationships between predictor and outcome variables. Future studies incorporating a longitudinal design will enable a more reliable test of the relationship between patient-perceived PCS and patient treatment adherence than was possible in the present study. Another study limitation is that the 67 participating health care sites from across the country were not randomly selected. Given the difficulty in recruiting such a large number of health care sites (as well as our interest in collecting data from Latino patients who are often underrepresented in health care research), multiple recruitment strategies were necessary, including the snowball technique of having participating sites help recruit other sites to be participants. These recruitment strategies resulted in a large number of sites from urban settings in large states. Although the diversity of sites is a strength of this study, this design does not allow for evaluating the role of health care site in the constructs of interest. Future research with a focus on specific health care context can help to clarify the role of health care site in the relations examined in the present study.

It is also noteworthy that the present study only examined a few of the possible cultural and language influences in patient-provider interactions involving Latino patients as predictors of treatment adherence among these patients. Future studies similar to the present study should include assessment of and control for other influences in patient-provider interactions involving Latino patients. Examples of such other variables are values that are important in the Latino culture such as respect for authority, collectivism (vs. individualism), and spirituality, as well as observed stigma and poor language-based communication fidelity.

While the revised PC-CSHC model tested in this study includes linkages that have been found to be significant in research with culturally diverse adult patients, including Latino patients, the model does not capture all of the Latino-specific cultural constructs related to Latino patient treatment adherence. Specifically, cultural factors, including *familismo*, *fatalismo*, *machismo*, *personalismo*, and *simpatia* may be particularly important to consider in research that aims to better understand the behaviors and attitudes associated with treatment adherence among Latino patients [36]. Future research should consider how these Latino-specific cultural constructs may be captured through the existing constructs of the revised PC-CSHC model and/or could be added to more fully capture Latino patient treatment adherence.

Finally, a noteworthy limitation of this study is the low Cronbach’s alpha scores for the measures of treatment adherence and patient satisfaction (i.e., the GAM and the general satisfaction subscale of the PSQ-18). Specifically, the Cronbach’s alphas for these were lower than desirable, <70 (Table 1). Consequently, the results of the current study should be interpreted with caution; however, it is important to note that lower alpha scores are typical when conducting minority inclusive research with these measures [37, 38].

### Implications

The findings from the present study highlight the need for interventions aimed at improving communication between health care providers and patients, such as through the use of interpreters. These interventions may not only increase the rate of adherence to general treatment recommendations among Spanish-preferred Latino patients but may also improve their trust in their providers and their satisfaction with the provider care experienced. Opportunities for Latino patients to identify and communicate what promotes their trust in and satisfaction with health care received could result in needed input to inform such interventions.

Increasing the number of Latino health care providers may also increase the pool of linguistically and culturally sensitive providers available to treat Spanish-speaking patients, particularly those who prefer to speak Spanish. It is also possible that non-Latino health care providers can achieve sufficiently high levels of Spanish language proficiency and cultural sensitivity to achieve optimal rates of treatment adherence by their Spanish-preferred patients. Doing so will require that medical education emphasize the development of language skills in Spanish, and skills in cross-cultural communication among all students and residents [39].

The implications of the present study for health care sites is that these sites should indeed include patient centeredness (i.e., one component of patient-perceived cultural sensitivity in health care experienced) as a key way to improve quality of care as asserted by the Institute of Medicine [40]. Additionally, administrators at health care sites that predominately serve Spanish-speaking Latino patients need to assess the level of patient-perceived provider cultural sensitivity that is demonstrated by the providers at their site.
